# Vector-Borne Transmission of the Zika Virus Asian Genotype in Europe

**DOI:** 10.3390/v12030296

**Published:** 2020-03-09

**Authors:** Guillaume A. Durand, Géraldine Piorkowski, Laurence Thirion, Laetitia Ninove, Sandra Giron, Christine Zandotti, Jessica Denis, Cyril Badaut, Anna-Bella Failloux, Gilda Grard, Isabelle Leparc-Goffart, Xavier de Lamballerie

**Affiliations:** 1 National Reference Laboratory for Arboviruses, Institut de Recherche Biomédicale des Armées, 13010 Marseille, France; jessica.d03@orange.fr (J.D.); cbadaut@gmail.com (C.B.); gilda.grard@inserm.fr (G.G.); isabelle.leparcgoffart@gmail.com (I.L.-G.); 2 Unité des Virus Emergents (UVE: Aix-Marseille Univ–IRD 190–Inserm 1207–IHU Méditerranée Infection), 13010 Marseille, France; geraldine.piorkowski@inserm.fr (G.P.); laurence.thirion@ird.fr (L.T.); laetitia.ninove@ap-hm.fr (L.N.); christine.zandotti@ap-hm.fr (C.Z.); xavier.de-lamballerie@univ-amu.fr (X.d.L.); 3 French National Public Health Agency (Santé publique France), 13002 Marseille, France; sandra.giron@santepubliquefrance.fr; 4 Unité de Biothérapies anti-Infectieuses et Immunité, Institut de Recherche Biomédicale des Armées, 91220 Bretigny sur Orge, France; 5 Department of Virology, Arboviruses and Insect Vectors, Institut Pasteur, 75015 Paris, France; anna-bella.failloux@pasteur.fr

**Keywords:** arbovirus, zika, France, *Aedes albopictus*, autochthonous transmission, genotyping

## Abstract

Three autochthonous cases of Zika virus occurred in southern France in August 2019. Diagnosis relied on serology and transcription-mediated amplification. Attempts for virus isolation and ZIKV genome RT-PCR detection remained negative. Since the index case was not identified, we addressed the issue of genotyping and geographical origin by performing hemi-nested PCR and sequencing in the Pr gene. Analysis of 16 genotype-specific Single Nucleotides Polymorphisms identified the Asian genotype and suggested a Southeast Asia origin.

## 1. Introduction

The first vector-borne transmission of Zika virus (ZIKV) in Europe has been reported recently [1]. It occurred during August 2019 in the Mediterranean coastal department of Var (city of Hyeres) within an area of permanent implantation of *Aedes albopictus*, which represents the only potential vector locally. During the first steps of investigation, a small cluster of three cases that occurred in August 2019 was discovered retrospectively (in October 2019) within a circle with a radius 50 meters. The index case was not identified and the issue of the geographical origin and genetic typing of the virus strain remained unanswered. This question is of specific importance since recent vector competence studies suggested that *Ae. albopictus* from France can transmit more efficiently the African genotype of ZIKV than the Asian genotype [2]. The latter includes both viruses that have been circulating in Asia for decades (as far as is known without causing large epidemics), and viruses that circulated during the recent important outbreaks in the Americas (mostly Latin America and the Caribbean) [3,4]. The aim of the current study was to determine the ZIKV genotype implicated in cases of local transmission in France, 2019.

## 2. Standard Laboratory Investigations

The first case, a 52-year-old woman, and then two additional cases, respectively a 47-year-old man and a 62-year-old woman, were identified using serological techniques. All cases were neighbours living in the same area (<100 m radius); they did not know each other. Neither cases nor sexual partners were found to have travelled outside the city within the 15 days before the onset of symptoms. Serology performed on the children and partners of the cases were negative. No Asian shop or warehouse receiving stock from Asia were found in this residential area. Earlier samples were tested by molecular approaches, whereas later ones were tested using in-house ELISA with a whole precipitated and inactivated virus, Zika EDIII recombinant protein (IgM and IgG antibodies to ZIKV), and confirmed by virus neutralization test. All cases were found positive in ELISA, Zika EDIII-based ELISA and virus neutralization, and negative for neutralizing antibodies against West-Nile and Dengue serotype 1 to 4 viruses [5]. All attempts for virus isolation and detection of the ZIKV genome by RT-PCR (RealStar Zika Virus RT-PCR kit, Altona Diagnostics, Germany [6]) in serum, urine and semen were negative, even in early samples collected less than two days after onset. This was presumably because of a conjunction of unfavourable circumstances including initial low viral loads, iterative freeze–thaw cycles and suboptimal conservation temperatures.

## 3. TMA, Nested PCR and Sequencing Strategy

**Figure 1:**
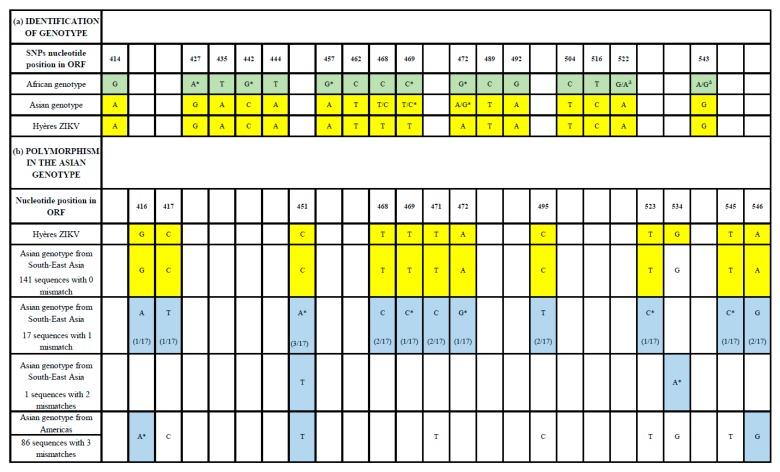
Identification of the genotype (**a**) and analysis of polymorphism in the Asian genotype (**b**). * Non-synonymous mutations. Δ Present only in sequence KY288905.

This situation rendered usual sequencing techniques inappropriate and, with the constraint of limited availability of biological resources, led to imagining a different typing strategy:

1. We used the extremely sensitive Transcription-Mediated Amplification (TMA) technology (Hologic, Marlborough, Massachusetts, United States [7]) to identify samples in which residual amounts of the ZIKV RNA genome could be detected from serum.
2. We performed in silico sequence analysis to identify genomic regions in which genotype-specific single nucleotide polymorphisms (SNP) would allow a clear distinction between the African and Asian genotypes of ZIKV. Such SNPs are present in different parts of the genome, but the Pr gene is of specific interest because it includes 16 SNPs, which individually strictly discriminate between the Asian and African genotype (see Figure 1a). It is therefore expected that sequencing this region would allow the unambiguous identification of the genotype incriminated. We designed a hemi-nested PCR set of primers allowing the amplification of a 139-nt sequence (primers excluded), including these SNPs (Supplementary Materials). All amplifications were performed in quadruplicate in the presence of 10 (PBS) negative controls. When tested, electrophoresis onto agarose gels of secondary PCR products revealed a band corresponding to the expected size in Case 3 from Day 1’s sample for three replicates, whereas PBS controls remained negative.
3. We used the secondary PCR reaction of all tested samples and negative controls for NGS sequencing using the S5 Ion Torrent technology (ThermoFisher Scientific, Waltham, MA, United States) [8]. Details are provided in the Supplementary Materials.

## 4. Genotyping and Probable Geographical Origin of the Strain

Sequence analysis identified ZIKV-specific reads for three of the four replicates from Case 3, and none for the other samples and the negative controls. A nucleotide Blast analysis of the consensus sequence was performed versus GenBank. Blastn was performed on 250 sequences; 245 sequences were conserved (excluding three synthetics, according to their titles names, and two duplicated sequences) for further examination. Figure 1b presents the results obtained for these 245 more closely related sequences. Sequence analysis showed that all 16 SNPs were specific of the Asian genotype. Further examination revealed that 141 sequences in GenBank were 100% identical with Case 1’s sequence, and all of them corresponding to ZIKV strains from Southeast Asia; 17 sequences from the same geographical region had one nucleotide mismatch and one had two mismatches. By contrast, 86 sequences from the American strains displayed three or more mismatches.

## 5. Discussion

In conclusion, the strain implicated in ZIKV autochthonous vector-borne transmission in the Var region belonged to the Asian genotype and was most probably imported from Southeast Asia (and not from the Americas). This was unexpected since previous vector competence studies found that both “Asian” and “American” strains belonging to the Asian genotype of ZIKV were poorly transmitted by French *Ae. albopictus* [2]. We propose several hypotheses that may allow reconciling these experimental and epidemiological findings.

First, the actual transmission potential of the incriminated ZIKV strain by local *Ae. albopictus* may be higher than suggested by previous competence studies. The mosquitoes used in these studies (collected in Corsica (2017) and Montpellier (2018)) may not adequately represent the populations implanted in Hyères. This may be investigated by renewing these experiments using mosquitoes collected at the precise location where autochthonous transmission occurred. Similarly, the transmission potential of the Asian strain used in competence studies (namely ZIKV Cambodia isolated from a human case in 2010 (FSS 13025, Asian genotype, passage 3, GenBank reference: KU955593)) may be different from that of the Hyères strain. This strain includes one mismatch in the sequence described above, which is associated with a L/M amino-acid substitution. However, in the absence of isolation and complete genomic sequencing of this strain, it will be difficult to further investigate this item.

Second, environmental conditions may have contributed to allow local transmission despite the limited vector competence suggested by the limited amplitude of the epidemic event. Some inhabitants in the area of transmission reported unusually high vector densities, which epidemiological field investigations linked to the existence of an unmaintained pool that acted as a “mosquito factory”. The meteorological conditions in 2019 were quite specific: The peak temperature occurred early (in July vs. August usually) and was higher than the previous years; in addition, the summer months were very dry. Such conditions may have contributed to create specific transmission conditions, different from those observed under experimental conditions.

In conclusion, this study allowed identifying the genotype (Asian) and the most probable geographical origin (Southeast Asia) of the ZIKV strain implicated in the first Zika virus local vector-borne transmission in Europe. It shows that virus genotyping can be obtained from samples with low viral load by combining SNPs identification and NGS sequencing. It finally calls to a careful interpretation of experimental competence studies, which can indeed provide a comparative estimate of the transmission capacity associated to specific mosquito and virus strains, but reflect specific combinations of mosquitoes, viruses and environmental conditions.

## Supplementary Materials

The following are available online at https://www.mdpi.com/1999-4915/12/3/296/s1, File F1: Supplementary Materials.
