# Supplementary material for: Vector-Borne Transmission of the Zika Virus Asian Genotype in Europe
Source: Viruses. 2020 Mar 9;12(3):296. doi: 10.3390/v12030296 (PMC7150815; doi:10.3390/v12030296)
Supplement: Supplementary file 1 [file viruses-12-00296-s001.pdf]

Supplementary Materials

Extraction of nucleic acids

Nucleic acids were extracted from 140 µL of each samples using the QIAamp Viral RNA Mini Kit (Qiagen, Hilden, Germany) according to manufacturer's instructions (elution volume: 60 µL).

Primary RT-PCR

Primary RT-PCR was performed with the SuperScript III Taq High Fidelity DNA Polymerase Kit (Thermo Fisher Scientific, USA) with primers S1 and R2 and 3 µL of RNA template in final volume of 25 µL. The thermal profile used was 50°C (30 min), 94 °C (2 min) followed by 40 amplification cycles (94°C (30 sec), 50°C (30 sec), 68°C (1 min)) and a last step at 68 °C for 7 min.

S1: TGCATACTAYATGTACTTGGA (ORF positions 390-410)

R2: GAYGTCGTGTTGCACCARCAAT (ORF positions 563-584)

Nested PCRs

Nested PCRs were performed with the DreamTaq kit (Thermo Fisher Scientific) with S1 and R3 primers in a 50 µL reaction format including 5 µL of primary PCR solution. The cycling program consisted of 94 °C (5 min), followed by 40 cycles (94°C (30 sec), 50°C (45 sec), 72°C (1 min)) and a last step at 72°C for 7 min. All amplifications were performed in quadruplicate in the presence of 10 (PBS) negative controls on a SensoQuest Labcycler Gradient thermocycler.

R3: ACCARCAATCGACRTCATCTGG (ORF positions 390-410)

DNA amplicons were revealed by electrophoresis on 2% agarose gel with SYBR Safe DNA gel Stain (Invitrogen), with a molecular weight marker of 100-bp DNA ladder (Promega) and then visualized under UV illumination.

NGS sequencing

Sequencing was performed using the S5 Ion torrent technology (ThermoFisher Scientific). Briefly, samples were quantified using Qubit® dsDNA HS Assay Kit and Qubit 2.0 fluorometer (ThermoFisher Scientific). Libraries were built using AB Library Builder System (ThermoFisher Scientific) following manufacturer's instructions. It consists in the addition to DNA of barcode for sample identification, and primers. Following real time PCR quantification (Ion Library TaqMan™ Quantitation Kit, ThermoFisher Scientific), equimolar pools of libraries were realized. The emulsion PCR of the pools and loading on 520 chips were done using the automated Ion Chef instrument (ThermoFisher Scientific).

Sequencing was performed using the S5 Ion torrent technology (Thermo Fisher Scientific) following manufacturer's instructions. Consensus sequence was obtained using CLC genomics workbench software 12.0 (Qiagen, Hilden, Germany) after removing the thirty first and last nucleotides of each read, trimming reads depending on quality (reads with quality over >99%) and length (reads over 100 pb were kept) and mapping them on a reference (most similar sequence after Blastn). A *de novo* contig was also produced to ensure that the consensus sequence was not affected by the reference sequence. For negative controls, reads were mapped on the consensus sequence of ZIKV.

Autochthonous consensus sequence:

CAGAAGCGATGCTGGGGAGGCCATATCTTTTCCAACCACACTGGGGATGAATAAGTGTTA
TATACAGATCATGGATCTTGGACACATGTGTGATGCCACCATGAGCTATGAATGCCCTAT
GCTGGATGAGGGGGTAGAACCAGATGACGTCG

Blastn were realized on NCBI website tool to compare ZIKV sequence to the 224 more closely related sequences. Alignment were realized using MAFFT online tool (https://mafft.cbrc.jp/alignment/server/) and MEGA 6 software (Tamura, Stecher, Peterson, Filipski, and Kumar 2013).
